# Modifiable pathways for colorectal cancer: a mendelian randomisation analysis

**DOI:** 10.1016/S2468-1253(19)30294-8

**Published:** 2019-10-24

**Authors:** Alex J Cornish, Philip J Law, Maria Timofeeva, Kimmo Palin, Susan M Farrington, Claire Palles, Mark A Jenkins, Graham Casey, Hermann Brenner, Jenny Chang-Claude, Michael Hoffmeister, Iva Kirac, Tim Maughan, Stefanie Brezina, Andrea Gsur, Jeremy P Cheadle, Lauri A Aaltonen, Ian Tomlinson, Malcolm G Dunlop, Richard S Houlston

**Affiliations:** a Division of Genetics and Epidemiology, Institute of Cancer Research, London, UK; b Cancer Research UK Edinburgh Centre and Medical Research Council Human Genetics Unit, Institute of Genetics and Molecular Medicine, University of Edinburgh, Edinburgh, UK; c Edinburgh Cancer Research Centre, Institute of Genetics and Molecular Medicine, University of Edinburgh, Edinburgh, UK; d Medicum and Genome-Scale Biology Research Program, Research Programs Unit, Department of Medical and Clinical Genetics, University of Helsinki, Helsinki, Finland; e Gastrointestinal Cancer Genetics Laboratory, Institute of Cancer and Genomic Sciences, University of Birmingham, Birmingham, UK; f Centre for Epidemiology and Biostatistics, University of Melbourne, Melbourne, VIC, Australia; g Center for Public Health Genomics, University of Virginia, Virginia, VA, USA; h Division of Clinical Epidemiology and Aging Research, German Cancer Research Center, Heidelberg, Germany; i German Cancer Consortium, German Cancer Research Center, Heidelberg, Germany; j Division of Preventive Oncology, German Cancer Research Center, Heidelberg, Germany; k Unit of Genetic Epidemiology, German Cancer Research Center, Heidelberg, Germany; l Division of Preventive Oncology, National Center for Tumor Diseases, Heidelberg, Germany; m Cancer Epidemiology Group, University Medical Center Hamburg-Eppendorf, University Cancer Center Hamburg, Hamburg, Germany; n Department of Surgical Oncology, University Hospital for Tumours, Sestre milosrdnice University Hospital Centre, Zagreb, Croatia; o Department of Oncology, University of Oxford, Oxford, UK; p Institute of Cancer Research, Department of Medicine I, Medical University of Vienna, Vienna, Austria; q Institute of Medical Genetics, School of Medicine, Cardiff University, Cardiff, UK

## Abstract

**Background:**

Epidemiological studies have linked lifestyle, cardiometabolic, reproductive, developmental, and inflammatory factors to the risk of colorectal cancer. However, which specific factors affect risk and the strength of these effects are unknown. We aimed to examine the relationship between potentially modifiable risk factors and colorectal cancer.

**Methods:**

We used a random-effects model to examine the relationship between 39 potentially modifiable risk factors and colorectal cancer in 26 397 patients with colorectal cancer and 41 481 controls (ie, people without colorectal cancer). These population data came from a genome-wide association study of people of European ancestry, which was amended to exclude UK BioBank data. In the model, we used genetic variants as instruments via two-sample mendelian randomisation to limit bias from confounding and reverse causation. We calculated odds ratios per genetically predicted SD unit increase in each putative risk factor (OR_SD_) for colorectal cancer risk. We did mendelian randomisation Egger regressions to identify evidence of potential violations of mendelian randomisation assumptions. A Bonferroni-corrected threshold of p=1·3 × 10^−3^ was considered significant, and p values less than 0·05 were considered to be suggestive of an association.

**Findings:**

No putative risk factors were significantly associated with colorectal cancer risk after correction for multiple testing. However, suggestive associations with increased risk were noted for genetically predicted body fat percentage (OR_SD_ 1·14 [95% CI 1·03–1·25]; p=0·0086), body-mass index (1·09 [1·01–1·17]; p=0·023), waist circumference (1·13 [1·02–1·26]; p=0·018), basal metabolic rate (1·10 [1·03–1·18]; p=0·0079), and concentrations of LDL cholesterol (1·14 [1·04–1·25]; p=0·0056), total cholesterol (1·09 [1·01–1·18]; p=0·025), circulating serum iron (1·17 [1·00–1·36]; p=0·049), and serum vitamin B12 (1·21 [1·04–1·42]; p=0·016), although potential pleiotropy among genetic variants used as instruments for vitamin B12 constrains the finding. A suggestive association was also noted between adult height and increased risk of colorectal cancer (OR_SD_ 1·04 [95% CI 1·00–1·08]; p=0·032). Low blood selenium concentration had a suggestive association with decreased risk of colorectal cancer (OR_SD_ 0·85 [95% CI 0·75–0·96]; p=0·0078) based on a single variant, as did plasma concentrations of interleukin-6 receptor subunit α (also based on a single variant; 0·98 [0·96–1·00]; p=0·035). Risk of colorectal cancer was not associated with any sex hormone or reproductive factor, serum calcium, or circulating 25-hydroxyvitamin D concentrations.

**Interpretation:**

This analysis identified several modifiable targets for primary prevention of colorectal cancer, including lifestyle, obesity, and cardiometabolic factors, that should inform public health policy.

**Funding:**

Cancer Research UK, UK Medical Research Council Human Genetics Unit Centre, DJ Fielding Medical Research Trust, EU COST Action, and the US National Cancer Institute.

## Introduction

Colorectal cancer is the third most commonly diagnosed cancer and the second leading cause of cancer-related death in the world.^1^ It accounted for around 1·8 million new cases and 860 000 deaths in 2018.^1^ According to demographic trajectories, the yearly global burden of colorectal cancer is projected to increase to more than 3 million new cases and 1·6 million deaths by 2040.^1^ Differences in the incidence of colorectal cancer between countries and studies of international migration have suggested a role for dietary and other lifestyle factors in disease development.^2^ Thus, interest is increasing in the development of public health programmes to reduce the incidence of colorectal cancer by targeting modifiable risk factors.

The World Cancer Research Fund and the American Institute for Cancer Research have concluded that there is convincing evidence that body-mass index (BMI) and alcohol intake are causally associated with increased risk of colorectal cancer, and that physical activity is causally associated with reduced risk.^3^ They also concluded that red meat intake is probably causally associated with increased risk, whereas dietary fibre, dairy products, and calcium supplements are probably causally associated with reduced risk.^3^ For most other risk factors, however, evidence is too inconclusive to reliably establish causal associations.^3^

**Research in context**

**Evidence before this study**

We searched PubMed to identify dietary, lifestyle, obesity-related, inflammatory, reproductive, and developmental factors potentially affecting risk of colorectal cancer that had been assessed in observational epidemiological studies published in English up to Nov 30, 2018. The appendix 1 (p 1) includes a full list of search terms used. Studies provide strong evidence for an association between body-mass index and hypercholesterolaemia and increased risk of colorectal cancer. Evidence from conventional observational studies for most other factors is too inconclusive to reliably establish specific associations.

**Added value of this study**

Mendelian randomisation exploits germline genetic variants as instrumental variables for putative risk factors. Because these genetic variants are randomly assorted at conception, they are not affected by reverse causation and so can provide evidence for causal relationships. We used genetic variants for 39 potentially modifiable risk factors in 26 397 patients with colorectal cancer and 41 481 controls who did not have colorectal cancer. We identified suggestive evidence for associations between serum vitamin B12, iron, and selenium concentrations and colorectal cancer. In addition to providing suggestive evidence for a causal relationship between high body-mass index and other measures of obesity and increased colorectal cancer risk, we found evidence for an association between genetically predicted LDL cholesterol and increased colorectal cancer risk.

**Implications of all the available evidence**

Our analysis provides genetic corroboration of causal relationships between raised body-mass index, hypercholesterolaemia, and increased colorectal cancer risk. Our findings support the restriction of vitamin B12 supplementation to people with a known indication, such as proven deficiency, and highlight important targets for primary prevention of colorectal cancer, including lifestyle, obesity, and cardiometabolic factors.

Much of the available evidence for causal relationships between potentially modifiable factors and risk of colorectal cancer comes from observational studies,^3^ which are susceptible to confounding bias and reverse causation.^4^ Data from randomised trials tend to be scarce and are often inconclusive.^5^, ^6^ Furthermore, identification of which specific components of risk factors such as diet are important is notoriously problematic in conventional observational epidemiological studies.^7^ Mendelian randomisation is an analytic approach in which germline genetic variants are used as proxies, or instrumental variables, for putative risk factors.^8^ Because these genetic variants are randomly assorted at conception, they are not influenced by reverse causation, and, in the absence of pleiotropy (ie, associations between genetic variants and disease through alternative pathways), they can provide unconfounded estimates of disease risk.^8^ Mendelian randomisation analyses are increasingly used to examine the potential effects of interventions on disease risk because they circumvent many of the limitations of conventional observational studies.

In this study, we investigated potentially causal and modifiable risk factors for colorectal cancer by using a two-sample mendelian randomisation framework, in which genetic variants associated with relevant risk factors as instrumental variables were first identified from genome-wide association studies (GWAS). We then assessed the association between these instrumental variables and colorectal cancer in a large GWAS.

## Methods

### Identification of risk factors

In this two-sample mendelian randomisation analysis, we used genetic variants as instruments to examine the relationship between 39 potentially modifiable risk factors and colorectal cancer. The genetic variant data were from patients with colorectal cancer and controls (ie, people without colorectal cancer) who were recruited to GWASs of people of European ancestry before May, 2019.

We focused on potentially modifiable dietary, lifestyle, obesity-related, inflammatory, reproductive, and developmental factors that were discussed in association with the risk of colorectal cancer in a report by the World Cancer Research Fund and the American Institute for Cancer Research.^3^ We also searched PubMed to identify additional modifiable risk factors for colorectal cancer that have been reviewed in published epidemiological meta-analyses (up to Nov 30, 2018) or mendelian randomisation analyses (up to March 1, 2019; appendix 1 p 1; appendix 2 table S1). Ethical approval was not sought for this specific project because all data came from summary statistics of published GWAS, and no individual-level data were used.

Single-nucleotide polymorphisms (SNPs) associated with putative risk factor traits suitable for use in mendelian randomisation analyses were identified from the largest GWAS or meta-analysis of each trait done to date (appendix 2 table S2). Traits were only considered if the proportion of variance explained by the associated SNPs was greater than 0·1%. Estimates of the proportion of variance explained were either obtained directly from publications or computed directly from the association statistics.^9^ Suitable genetic instruments were not available for many risk factors, such as physical activity, dietary patterns, and vitamin C intake, which precluded their inclusion (appendix 2 table S1). We considered only continuous traits, because analysis of binary traits (eg, disease status) with binary outcomes in two-sample mendelian randomisation frameworks can result in inaccurate causal estimates.^10^ Only SNPs associated with each trait at p values of less than 5 × 10^−8^ in a GWAS of European populations with a minor allele frequency greater than 0·01 were considered as potential instruments. To mitigate against co-linearity between SNPs, which can bias causal effect estimates, we used MR-Base to exclude correlated SNPs at a linkage disequilibrium threshold of r^2^ greater than 0·01, and retained SNPs with the strongest effect on the associated trait.^11^

### Procedures

To examine the association of each genetic instrument with the risk of colorectal cancer, we used summary effect estimates and corresponding SEs from Law and colleagues' meta-analysis^12^ of 15 colorectal cancer GWAS. After imputation, this meta-analysis related more than 10 million genetic variants to colorectal cancer in people of European ancestry. UK BioBank data were used to obtain genetic instruments for age at menarche, basal metabolic rate, birthweight, body fat percentage, and waist circumference. These data were also used in one of the GWAS included in Law and colleagues' meta-analysis,^12^ so, to avoid bias caused by sample overlap,^13^ we excluded the GWAS in which UK BioBank data were used and recomputed association statistics for the remaining 14 studies (appendix 2 table S3) with an inverse variance-weighted fixed-effects model similar to that described by Law and colleagues.^12^

The resulting meta-analysis population comprised 26 397 patients with colorectal cancer and 41 481 controls. SNPs with poor imputation quality (ie, info score <0·8) were not included in the mendelian randomisation analysis. As some potentially modifiable reproductive risk factors are specific to women, when sex-specific data were available we further computed colorectal cancer association statistics in the 7952 female cases and 11 680 female controls. We used MR-Base to harmonise SNPs to ensure that the effect estimates of each SNP on each trait and colorectal cancer risk corresponded to the same allele.^11^ Effect estimates for the association of each trait SNP with colorectal cancer risk are in appendix 2 (table S2). For all vitamins studied (ie, vitamins A, B6, B12, and E), positive β values mean that the effect allele was associated with increased serum concentrations.

Fatty acid metabolism involves sequential enzymatic conversions (appendix 1 p 13), and SNPs that affect the metabolism of one fatty acid are therefore often associated with circulating concentrations of several fatty acids.^14^ Additionally, many genes involved in desaturation and elongation of fatty acids are constituents of numerous fatty acid pathways, and thereby affects circulating concentrations of several fatty acid classes (appendix 1 p 13). To limit bias introduced by such vertical and horizontal pleiotropy, we restricted our analysis to classes of fatty acids (such as omega-6 polyunsaturated fatty acids and monounsaturated fatty acids), rather than individual fatty acids, and excluded SNPs known to be associated with several fatty acid classes (appendix 2 table S7).

### Statistical analysis

**Figure 1:**
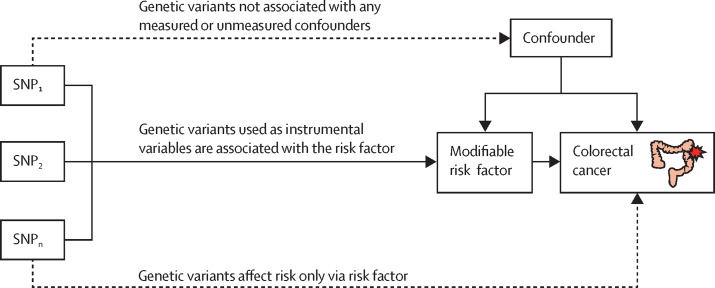
Principles of mendelian randomisation and assumptions that need to be satisfied to derive unbiased causal effect estimates Dashed lines represent direct causal and potential pleiotropic effects that could violate mendelian randomisation assumptions. SNP=single-nucleotide polymorphism.

Our mendelian randomisation methods were predicated on the assumption that genetic variants used as instruments for a risk factor are associated with the risk factor and not with a confounder or alternative causal pathway (figure 1). Additionally, to accurately estimate the size of the causal effect of the risk factor under investigation, the associations have to be linear and unaffected by statistical interactions.^15^ We used the Wald ratio to estimate causal effects for each SNP (appendix 1 pp 9–12). For traits for which more than one SNP was available as an instrument, causal effects were estimated with the random-effects maximum likelihood estimation method.^16^ To assess the robustness of our findings, we also obtained weighted median estimates^17^ and mode-based estimates.^18^ We used the MR-Egger regression approach to assess the extent to which directional pleiotropy could affect causal estimates.^19^ Finally, we did a leave-one-out analysis with the multiplicative random-effects inverse variance weighted method^11^ to examine the effect of outlying and pleiotropic SNPs on causal estimates (appendix 2 table S4).

*I*^2^ statistics were computed to estimate the proportion of variance across SNPs due to heterogeneity (appendix 2 table S5). Results are reported as odds ratios per genetically predicted standard deviation unit increase in each putative risk factor (OR_SD_) and 95% CIs. To address the issue of multiple testing, we applied a Bonferroni-corrected significance threshold, which was computed as 0·0013 (ie, 0·05/39 [for the 39 putative risk factors]). p values between 0·0013 and 0·05 were considered as suggestive of a potential association. The power of mendelian randomisation to show a causal effect depends on the proportion of variance in the risk factor explained by the genetic variants used as instruments, and we therefore estimated study power at an α of 0·05 for each risk factor a priori.^20^ Statistical analyses were done in R (version 3.4.0) and mendelian randomisation analyses were done in MR-Base.^11^

### Role of the funding source

The funders of the study had no role in study design, data collection, data analysis, data interpretation, or writing of the report. The corresponding author had full access to all the data in the study and had final responsibility for the decision to submit for publication.

## Results

The 39 potentially modifiable risk factors for colorectal cancer that we studied included 13 traits related to diet and lifestyle, three to fatty acid profile and metabolism, ten to obesity, five to lipids and lipid transport, three to inflammatory factors, three to sex hormones and reproduction, and two to developmental and growth factors. The number of SNPs used as genetic instruments for these potentially modifiable risk factors ranged from one to 2487.

**Figure 2:**
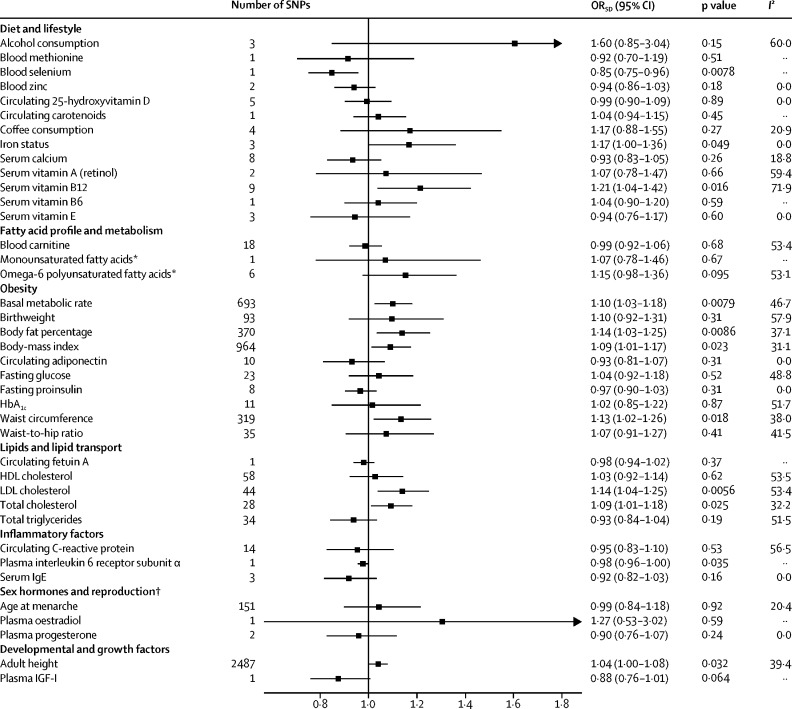
OR_SD_ for associations between genetically predicted risk factors and colorectal cancer We used a maximum likelihood estimate random-effects method to summarise Wald ratio estimates from individual SNPs. OR_SD_=odds ratio per genetically predicted SD unit increase in the risk factor. SNP=single-nucleotide polymorphism. *Restricted analyses that excluded SNPs associated with other classes of fatty acid. †OR_SD_ estimated based on colorectal cancer summary statistics for women only.

With regard to the diet and lifestyle factors investigated, under a random-effects maximum likelihood estimation model, we noted a suggestive association between genetically predicted high serum vitamin B12 concentrations and increased risk of colorectal cancer (OR_SD_ 1·21 [95% CI 1·04–1·42]; p=0·016; figure 2). However, there was substantial heterogeneity between the SNPs used as instrumental variables for this association (*I*^2^ 71·9). Leave-one-out analysis showed that SNP rs602662 at a known risk locus for colorectal cancer had a strong influence on the causal estimate for serum vitamin B12 concentrations (appendix 2 table S4).^12^ There was a suggestive association between genetically predicted increased serum iron concentrations and increased risk of colorectal cancer (OR_SD_ 1·17 [95% CI 1·00–1·36]; p=0·049), with no outlying genetic variant identified (appendix 1 pp 9–12). We also noted a suggestive association between raised serum selenium concentrations and decreased colorectal cancer risk (OR_SD_ 0·85 [95% CI 0·75–0·96]; p=0·0078), but this association was based on only one SNP. Genetically predicted alcohol and coffee consumption, and blood concentrations of methionine, zinc, 25-hydroxyvitamin D, carotenoids, calcium, and vitamins A, B6, and E were not associated with risk of colorectal cancer (figure 2). Causal effect estimates for serum vitamin B12 concentration were similar in sensitivity analyses in which we used weighted median and mode-based methods (appendix 2 table S5). MR-Egger regression showed no evidence of directional pleiotropy in the analyses of vitamin B12 or serum iron concentration (appendix 2 table S6). The causal effects estimated by MR-Egger were not significant for vitamin B12 (appendix 2 table S5).

In our restricted analysis of fatty acid profile and metabolism, no association was noted between risk of colorectal cancer and omega-6 polyunsaturated or monounsaturated fatty acids concentrations or blood concentrations of the fatty acid transport molecule carnitine (figure 2). After removal of potentially pleiotropic SNPs, only one SNP was suitable for use as an instrumental variable for monounsaturated fatty acids concentration, which meant that sensitivity analysis could not be done.

When we included information about all genetic variants associated with cardiometabolic factors, measures of obesity and hyperlipidaemia were suggestively associated with colorectal cancer (figure 2). Specifically, suggestive associations were noted between genetically predicted basal metabolic rate (OR_SD_ 1·10 [95% CI 1·03–1·18]; p=0·0079), body fat percentage (1·14 [1·03–1·25]; p=0·0086), BMI (1·09 [1·01–1·17]; p=0·023) and waist circumference (1·13 [1·02–1·26]; p=0·018) and increased risk of colorectal cancer. We noted no association between birthweight or circulating adiponectin concentrations and risk of colorectal cancer (figure 2). Causal estimates for basal metabolic rate, BMI, and waist circumference were broadly concordant in weighted median and mode-based sensitivity analyses (appendix 2 table S5). Conversely, the effect estimate for body fat percentage from the mode-based estimate approach was not significantly associated with colorectal cancer risk (appendix 2 table S5), suggesting that some of the instruments used to assess the causal effects of body fat percentage might have been invalid. MR-Egger regression did not identify evidence of horizontal pleiotropy for body fat percentage or any other obesity-related trait (appendix 2 table S6).

Genetically predicted LDL cholesterol (OR_SD_ 1·14 [95% CI 1·04–1·25]; p=0·0056) and total cholesterol (1·09 [1·01–1·18]; p=0·025) were suggestively associated with increased risk of colorectal cancer. No association between HDL cholesterol or total triglyceride concentrations and colorectal cancer risk was detected (figure 2). Similarly, genetically predicted metrics of glycaemia were not associated with colorectal cancer risk (figure 2).

A suggestive association based on one SNP was noted between plasma concentrations of interleukin 6 receptor subunit α and decreased risk of colorectal cancer (OR_SD_ 0·98 [95% CI 0·96–1·00]; p=0·035). Associations between circulating C-reactive protein and serum IgE and colorectal cancer risk were not identified (figure 2).

We noted no association between age at menarche, a surrogate for endogenous oestrogen exposure, and risk of colorectal cancer (OR_SD_ 0·99 [95% CI 0·84–1·18]; p=0·92) in women. Similarly, we did not note associations between plasma oestradiol and progesterone concentrations and colorectal cancer (figure 2). The genetic variants used as instruments for these traits explain only a small proportion of their variance (table), and we were therefore unable to exclude a small-to-moderate effect of sex hormone exposure on colorectal cancer risk. MR-Egger regression analysis of genetic instruments for age at menopause provided evidence of horizontal pleiotropy (p=0·01; appendix 2 table S6) and we therefore did not consider this trait in our mendelian randomisation analysis.

**Table:** Modifiable risk factors for colorectal cancer included in mendelian randomisation analysis

	**PubMed identifier**	**SNPs used in mendelian randomisation analysis**	**Proportion of variance explained by SNPs**	**Power to identify OR**_SD_**of 0·91 or 1·10**	**Power to identify OR**_SD_**of 0·75 or 1·33**	**F statistic**
**Diet and lifestyle**						
Alcohol consumption	28 937 693	3	0·002	0·082	0·364	66·73
Blood methionine	24 816 252	1	0·004	0·124	0·676	30·57
Blood selenium	23 720 494	1	0·020	0·417	1·000	114·36
Blood zinc	23 720 494	2	0·046	0·746	1·000	62·58
Circulating 25-hydroxyvitamin D	29 343 764	5	0·026	0·512	1·000	431·37
Circulating carotenoids	19 185 284	1	0·028	0·531	1·000	106·36
Coffee consumption	25 288 136	4	0·005	0·147	0·788	124·16
Iron status	25 352 340	3	0·012	0·260	0·981	190·40
Serum calcium	24 068 962	8	0·026	0·503	1·000	202·32
Serum vitamin A (retinol)	21 878 437	2	0·007	0·175	0·879	34·69
Serum vitamin B12	23 754 956	9	0·047	0·760	1·000	252·08
Serum vitamin B6	19 303 062	1	0·014	0·307	0·994	26·67
Serum vitamin E	21 729 881	3	0·007	0·167	0·857	10·92
**Fatty acid profile and metabolism**						
Blood carnitine	24 816 252	18	0·139	0·995	1·000	65·81
Monounsaturated fatty acids*	27 005 778	1	0·003	0·097	0·493	36·29
Omega-6 polyunsaturated fatty acids*	27 005 778	6	0·024	0·477	1·000	55·68
**Obesity**						
Basal metabolic rate	30 305 743	693	0·122	0·990	1·000	66·11
Birthweight	30 305 743	93	0·025	0·487	1·000	52·72
Body fat percentage	30 305 743	370	0·053	0·806	1·000	50·28
Body-mass index	30 124 842	964	0·079	0·929	1·000	60·69
Circulating adiponectin	22 479 202	10	0·018	0·372	0·999	65·12
Fasting glucose	22 581 228	23	0·036	0·639	1·000	93·73
Fasting proinsulin	20 081 858	8	0·061	0·858	1·000	87·33
Glycated haemoglobin A_1c_	20 858 683	11	0·018	0·381	0·999	78·72
Waist circumference	30 305 743	319	0·047	0·754	1·000	51·68
Waist-to-hip ratio	25 673 412	35	0·018	0·369	0·999	57·66
**Lipids and lipid transport**						
Circulating fetuin-A	28 379 451	1	0·143	0·996	1·000	1331·92
HDL cholesterol	24 097 068	58	0·061	0·856	1·000	105·31
LDL cholesterol	24 097 068	44	0·079	0·930	1·000	182·74
Total cholesterol	27 005 778	28	0·095	0·964	1·000	80·05
Total triglycerides	24 097 068	34	0·061	0·857	1·000	180·23
**Inflammatory factors**						
Circulating C-reactive protein	21 300 955	14	0·036	0·640	1·000	220·09
Plasma interleukin-6 receptor subunit α	29 875 488	1	0·604	1·000	1·000	5038·85
Serum immunoglobulin E	22 075 330	3	0·016	0·342	0·997	79·70
**Sex hormones and reproduction**†						
Age at menarche	30 305 743	151	0·048	0·303	0·993	58·11
Plasma oestradiol	26 014 426	1	0·011	0·105	0·553	31·47
Plasma progesterone	26 014 426	2	0·035	0·235	0·965	52·44
**Developmental and growth factors**						
Adult height	30 124 842	2487	0·380	1·000	1·000	171·41
Plasma IGF-I	29 875 488	1	0·014	0·314	0·995	48·51

The F statistic was used as a measure of potential weak instrument bias, with a low statistic (ie, <10) indicative of possible bias. SNPs=single-nucleotide polymorphisms. OR_SD_=odds ratio per genetically predicted SD unit increase in risk factor.

* Restricted analyses that excluded SNPs associated with other classes of fatty acids.

† OR_SD_ estimated based on colorectal cancer summary statistics for women only.

Although height is not modifiable once stabilised in adulthood, it is affected by developmental factors and growth processes, which might be modifiable. In concordance with evidence reviewed by the World Cancer Research Fund and the American Institute for Cancer Research,^3^ we noted a suggestive association between genetically predicted adult height and increased odds of colorectal cancer (OR_SD_ 1·04 [95% CI 1·00–1·08]; p=0·032), further supporting the notion that childhood factors affect subsequent disease risk. We noted no association between plasma IGF-1 concentrations and risk of colorectal cancer (figure 2). However, this analysis was based on only one genetic variant that accounts for only a small proportion of IGF-1 variance, and therefore had little power to detect an effect (table).

F statistics were high (>10) for all considered traits (table), but some of our findings might have been affected by weak instrument bias. For 19 of the traits that were not associated with colorectal cancer risk, our study had less than 80% power to identify OR_SD_ less than 0·91 or >1·10 (table), and we therefore cannot exclude the possibility that these traits have a small effect on disease risk.

## Discussion

This mendelian randomisation study, in which we used genetic variants as proxies for putative risk factors, provides suggestive evidence for associations between increased body fat percentage, BMI, waist circumference, basal metabolic rate, adult height, serum vitamin B12 concentrations, serum iron concentrations, LDL cholesterol, and total cholesterol and increased colorectal cancer risk. There was also suggestive evidence for possible associations between serum selenium and interleukin 6 receptor subunit α concentrations and decreased colorectal cancer risk.

Strengths of this study include that we examined multiple factors in relation to colorectal cancer risk by exploiting data from large GWAS. Many of the putative risk factors considered in this study have not previously been assessed with mendelian randomisation frameworks (appendix 2 table S8). Of the factors for which suggestive associations were identified, body fat percentage, waist circumference, basal metabolic rate, iron status, and blood selenium, serum vitamin B12, and plasma interleukin 6 subunit α concentrations have not previously been included in mendelian randomisation analyses of colorectal cancer risk. Even for risk factors that were included in previous analyses,^21^ the number of cases and controls in our analysis affords us greater power to detect causal associations with colorectal cancer and allows us to more accurately estimate effect magnitudes. For example, whereas Rodriguez-Broadbent and colleagues^22^ did not identify a significant association between LDL cholesterol and risk of colorectal cancer (OR_SD_ 1·05 [95% CI 0·92–1·18]; p=0·49), we identified a suggestive relationship (1·14 [1·04–1·25]; p=0·0056), possibly because our study had increased power. By comparing the results of this study to those of previous mendelian randomisation analyses of colorectal cancer risk, we could also identify previously reported causal relationships that might be false positives—eg, the previously reported association between genetically predicted C-reactive protein concentrations and colorectal cancer risk.^23^

As with all mendelian randomisation studies, excluding pleiotropy or an alternative direct causal pathway as the basis of the association was a challenge. High *I*^2^ statistics for many traits suggest that pleiotropy was present in this analysis. To address this issue, we did sensitivity analyses with the weighted median and model-based estimate methods, which can provide unbiased causal effect estimates even when many of the genetic variants used represent invalid instruments.^17^, ^18^ For most of the traits with either a significant or suggestive association with colorectal cancer risk, the effects estimated were similar in our sensitivity analyses, supporting causal relationships. Differences in causal effect estimates from the random-effects maximum likelihood estimation model and MR-Egger regression are possibly a result of the reduced power of MR-Egger regression to detect causal effects.^19^ Importantly, there is overlap between the cases and controls in this study, and those included in previous mendelian randomisation analyses,^21^ and results from this study cannot therefore be considered to be independent replication.

We found no evidence for an association between genetically predicted fasting glucose and proinsulin and risk of colorectal cancer, suggesting that metabolic syndrome might not influence risk through these factors. However, because of the limited power of this analysis, we cannot preclude these factors having small effects on colorectal cancer risk. Our estimate that an increase in adult height of one SD increases colorectal cancer risk by 4% is concordant with the findings of many observational studies.^3^ Increased exposure to growth hormones and insulin-like growth factors during childhood have been posited as potential mechanisms for this association.^24^ Although we did not identify a significant association between plasma IGF-1 concentrations and colorectal cancer risk, the limited power of this analysis means that we cannot rule out small-to-moderate effect sizes. Taller adults tend to have larger colons than do shorter adults, and thus larger populations of at-risk cells might also explain the apparent causal inference.

Our findings of an association between genetically predicted vitamin B12 concentrations and colorectal cancer risk are concordant with those of a randomised trial^25^ in which vitamin B12 supplementation was associated with increased risk. Although the associations were weaker than that for vitamin B12, we also found suggestive evidence to support high selenium concentrations having a beneficial effect and high iron concentrations a detrimental effect.

Further research is required to decipher the biological pathways underpinning associations. However, irrespective of the exact functional basis of associations identified via a genetic approach, our analysis highlights important targets for primary prevention of colorectal cancer in the population. First, the suggestive association between obesity and colorectal cancer risk suggests that reducing the population incidence of obesity is a priority for cancer prevention. Second, our findings suggest that hypercholesterolemia is causally linked to colorectal cancer risk and therefore support the hypothesis that increasing use of statins for prevention of cardiovascular disease could also reduce the burden of colorectal cancer. The limited power of this study to robustly define the relaionship between some putative risk factors and colorectal cancer provides the impetus for larger mendelian randomisation studies, which could elucidate relationships for the spectrum of colorectal neoplasia. Such work could shed additional light on other potentially modifiable factors that could then be targeted to reduce the overall burden of colorectal cancer.

## Data sharing

Genetic instruments can be obtained through MR-Base or from the individual referenced papers.
