# Supplementary material for: Modifiable pathways for colorectal cancer: a mendelian randomisation analysis
Source: Lancet Gastroenterol Hepatol. 2019 Oct 24;5(1):55–62. doi: 10.1016/S2468-1253(19)30294-8 (PMC7026696; doi:10.1016/S2468-1253(19)30294-8)
Supplement: Supplementary appendix 1 [file mmc1.pdf]

THE LANCET
Gastroenterology & Hepatology

Supplementary appendix

This appendix formed part of the original submission and has been peer reviewed. We post it as supplied by the authors.

Supplement to: Cornish AJ, Law PJ, Timofeeva M, et al. Modifiable pathways for colorectal cancer: a mendelian randomisation analysis. *Lancet Gastroenterol Hepatol* 2019; published online Oct 23. http://dx.doi.org/10.1016/S2468-1253(19)30294-8.

Modifiable pathways for colorectal cancer: A Mendelian randomisation analysis

SUPPLEMENTARY METHODS

Identification of potentially modifiable risk factors

To identify epidemiological meta-analyses of colorectal cancer (CRC) risk factors we searched PubMed with the terms: '((((colorectal cancer) OR colon cancer) OR rectal cancer) AND risk factor) AND meta analysis', restricting our search to reviews from the previous five years (search conducted 30 November 2018). Mendelian randomisation (MR) analyses of CRC risk factors were identified by further searching PubMed with the terms: '(((colorectal cancer) OR colon cancer) OR rectal cancer) AND ((Mendelian randomization) OR Mendelian randomisation)' (search conducted 1 March 2019).

Genetic instruments for putative risk factors

We obtained instruments for two developmental and growth factor[1,2], three sex hormones and reproduction[3,4], three fatty acid (FA)[5,6], three inflammatory[2,7,8], five lipid[6,9,10], ten obesity[1,3,11-16], and 13 other diet and lifestyle-related traits[5,17-27].

The genetic architectures of smoking initiation and number of cigarettes smoked per day differ[28], and these traits therefore need to be considered separately in MR analyses. Smoking initiation is a binary trait and was therefore not included, as analysis of binary exposures with binary outcomes using two-sample MR frameworks can result in inaccurate causal estimates[29]. Smoking status data were not available for all CRC genome-wide association study (GWAS) individuals, and we were therefore also unable to include number of cigarettes smoked per day in this analysis.

For each SNP used as a genetic instrument, we obtained the per-allele effect estimate on the putative risk factor, the standard error (SE) of this estimate, and the effect and reference alleles from the corresponding GWA. We standardized effect estimates to represent the effect of each SNP on the trait in units of standard

deviation (SD). Association strengths of genetic instruments for each putative risk factor were quantified by the F-statistic, with F>10 considered indicative of a strong instrument[30].

A central assumption of MR is that SNPs used as instrumental variables (IVs) are associated with the outcome only through the exposure, and are not confounded by pleiotropy[31]. A number of genes, including *FADS1*, *FADS2* and *ELOVL2,* control the metabolism of multiple FAs, and SNPs at these loci are therefore associated with circulating concentrations of more than one FA[32,33]. Assessing the effect of pleiotropy on MR causal estimates using approaches such as MR-Egger, weighted median estimator (WME) and mode-based estimates (MBE), requires multiple SNPs to be used as IVs. As many FAs have only been associated with a single or small number of SNPs[33], it is not possible to use such methods, and we therefore restricted our analysis of FAs on CRC risk to limit potential bias introduced by pleiotropic SNPs.

FA metabolism involves sequential enzymatic conversions, and SNPs influencing the metabolism of one FA can therefore be associated with circulating concentrations of multiple FAs of the same class (*i.e.* vertical pleiotropy). To limit the effect of vertical pleiotropy, we therefore considered classes of FA (*i.e.* omega-3 polyunsaturated fatty acids [PUFAs], omega-6 PUFAs and monounsaturated fatty acids [MUFAs]), rather than individual FAs, in our primary analysis.

Many genes involved in FA desaturation and elongation, such as *FADS1* and *ELOVL2,* form parts of multiple FA pathways, and therefore influence the circulating concentrations of FAs from more than one class (*i.e.* horizontal pleiotropy). To limit the effects of horizontal pleiotropy, we therefore excluded SNPs known to be associated with multiple classes of FA. Such potentially pleiotropic SNPs were identified using genome-wide significant SNPs from four GWAS[6,32,34,35]. SNPs were excluded if they themselves were associated with multiple FA classes, were in linkage disequilibrium with a SNP associated with another FA class (r^2>0.01), or were

within 500kb of a SNP associated with another FA class. In our primary analysis we consider only SNPs not known to be associated with another class of FA.

References

1. Yengo L, Sidorenko J, Kemper KE, et al. Meta-analysis of genome-wide association studies for height and body mass index in approximately 700000 individuals of European ancestry. *Hum Mol Genet* 2018; **15**(20): 3641-9.

2. Sun BB, Maranville JC, Peters JE, et al. Genomic atlas of the human plasma proteome. *Nature* 2018; **558**(7708): 73-9.

3. Bycroft C, Freeman C, Petkova D, et al. The UK Biobank resource with deep phenotyping and genomic data. *Nature* 2018; **562**(7726): 203-9.

4. Ruth KS, Campbell PJ, Chew S, et al. Genome-wide association study with 1000 genomes imputation identifies signals for nine sex hormone-related phenotypes. *Eur J Hum Genet* 2016; **24**(2): 284-90.

5. Shin SY, Fauman EB, Petersen AK, et al. An atlas of genetic influences on human blood metabolites. *Nat Genet* 2014; **46**(6): 543-50.

6. Kettunen J, Demirkan A, Wurtz P, et al. Genome-wide study for circulating metabolites identifies 62 loci and reveals novel systemic effects of LPA. *Nat Commun* 2016; **7**: 11122.

7. Dehghan A, Dupuis J, Barbalic M, et al. Meta-analysis of genome-wide association studies in >80 000 subjects identifies multiple loci for C-reactive protein levels. *Circulation* 2011; **123**(7): 731-8.

8. Granada M, Wilk JB, Tuzova M, et al. A genome-wide association study of plasma total IgE concentrations in the Framingham Heart Study. *J Allergy Clin Immunol* 2012; **129**(3): 840-5 e21.

9. Jensen MK, Jensen RA, Mukamal KJ, et al. Detection of genetic loci associated with plasma fetuin-A: a meta-analysis of genome-wide association studies from the CHARGE Consortium. *Hum Mol Genet* 2017; **26**(11): 2156-63.

10. Willer CJ, Schmidt EM, Sengupta S, et al. Discovery and refinement of loci associated with lipid levels. *Nat Genet* 2013; **45**(11): 1274-83.

11. Dastani Z, Hivert MF, Timpson N, et al. Novel loci for adiponectin levels and their influence on type 2 diabetes and metabolic traits: a multi-ethnic meta-analysis of 45,891 individuals. *PLoS Genet* 2012; **8**(3): e1002607.

12. Manning AK, Hivert MF, Scott RA, et al. A genome-wide approach accounting for body mass index identifies genetic variants influencing fasting glycemic traits and insulin resistance. *Nat Genet* 2012; **44**(6): 659-69.

13. Dupuis J, Langenberg C, Prokopenko I, et al. New genetic loci implicated in fasting glucose homeostasis and their impact on type 2 diabetes risk. *Nat Genet* 2010; **42**(2): 105-16.

14. Soranzo N, Sanna S, Wheeler E, et al. Common variants at 10 genomic loci influence hemoglobin A(1)(C) levels via glycemic and nonglycemic pathways. *Diabetes* 2010; **59**(12): 3229-39.

15. Shungin D, Winkler TW, Croteau-Chonka DC, et al. New genetic loci link adipose and insulin biology to body fat distribution. *Nature* 2015; **518**(7538): 187-96.

16. Strawbridge RJ, Dupuis J, Prokopenko I, et al. Genome-wide association identifies nine common variants associated with fasting proinsulin levels and provides new insights into the pathophysiology of type 2 diabetes. *Diabetes* 2011; **60**(10): 2624-34.

17. Clarke TK, Adams MJ, Davies G, et al. Genome-wide association study of alcohol consumption and genetic overlap with other health-related traits in UK Biobank (N=112 117). *Mol Psychiatry* 2017; **22**(10): 1376-84.

18. Evans DM, Zhu G, Dy V, et al. Genome-wide association study identifies loci affecting blood copper, selenium and zinc. *Hum Mol Genet* 2013; **22**(19): 3998-4006.

19. Jiang X, O'Reilly PF, Aschard H, et al. Genome-wide association study in 79,366 European-ancestry individuals informs the genetic architecture of 25-hydroxyvitamin D levels. *Nat Commun* 2018; **9**(1): 260.

20. Ferrucci L, Perry JR, Matteini A, et al. Common variation in the beta-carotene 15,15'-monooxygenase 1 gene affects circulating levels of carotenoids: a genome-wide association study. *Am J Hum Genet* 2009; **84**(2): 123-33.

21. Coffee and Caffeine Genetics Consortium, Cornelis MC, Byrne EM, et al. Genome-wide meta-analysis identifies six novel loci associated with habitual coffee consumption. *Mol Psychiatry* 2015; **20**(5): 647-56.

22. Benyamin B, Esko T, Ried JS, et al. Novel loci affecting iron homeostasis and their effects in individuals at risk for hemochromatosis. *Nat Commun* 2014; **5**: 4926.

23. O'Seaghdha CM, Wu H, Yang Q, et al. Meta-analysis of genome-wide association studies identifies six new Loci for serum calcium concentrations. *PLoS Genet* 2013; **9**(9): e1003796.

24. Mondul AM, Yu K, Wheeler W, et al. Genome-wide association study of circulating retinol levels. *Hum Mol Genet* 2011; **20**(23): 4724-31.

25. Grarup N, Sulem P, Sandholt CH, et al. Genetic architecture of vitamin B12 and folate levels uncovered applying deeply sequenced large datasets. *PLoS Genet* 2013; **9**(6): e1003530.

26. Tanaka T, Scheet P, Giusti B, et al. Genome-wide association study of vitamin B6, vitamin B12, folate, and homocysteine blood concentrations. *Am J Hum Genet* 2009; **84**(4): 477-82.

27. Major JM, Yu K, Wheeler W, et al. Genome-wide association study identifies common variants associated with circulating vitamin E levels. *Hum Mol Genet* 2011; **20**(19): 3876-83.

28. Tobacco and Genetics Consortium. Genome-wide meta-analyses identify multiple loci associated with smoking behavior. *Nat Genet* 2010; **42**(5): 441-7.

29. Disney-Hogg L, Cornish AJ, Sud A, et al. Impact of atopy on risk of glioma: a Mendelian randomisation study. *BMC Med* 2018; **16**(1): 42.

30. Lawlor DA, Harbord RM, Sterne JA, Timpson N, Davey Smith G. Mendelian randomization: using genes as instruments for making causal inferences in epidemiology. *Stat Med* 2008; **27**(8): 1133-63.

31. Davey Smith G, Hemani G. Mendelian randomization: genetic anchors for causal inference in epidemiological studies. *Hum Mol Genet* 2014; **23**(R1): R89-98.

32. Wu JH, Lemaitre RN, Manichaikul A, et al. Genome-wide association study identifies novel loci associated with concentrations of four plasma phospholipid fatty acids in the de novo lipogenesis pathway: results from the Cohorts for Heart and Aging Research in Genomic Epidemiology (CHARGE) consortium. *Circ Cardiovasc Genet* 2013; **6**(2): 171-83.

33. May-Wilson S, Sud A, Law PJ, et al. Pro-inflammatory fatty acid profile and colorectal cancer risk: A Mendelian randomisation analysis. *Eur J Cancer* 2017; **84**: 228-38.

34. Guan W, Steffen BT, Lemaitre RN, et al. Genome-wide association study of plasma N6 polyunsaturated fatty acids within the cohorts for heart and aging research in genomic epidemiology consortium. *Circ Cardiovasc Genet* 2014; **7**(3): 321-31.

35. Lemaitre RN, King IB, Kabagambe EK, et al. Genetic loci associated with circulating levels of very long-chain saturated fatty acids. *J Lipid Res* 2015; **56**(1): 176-84.

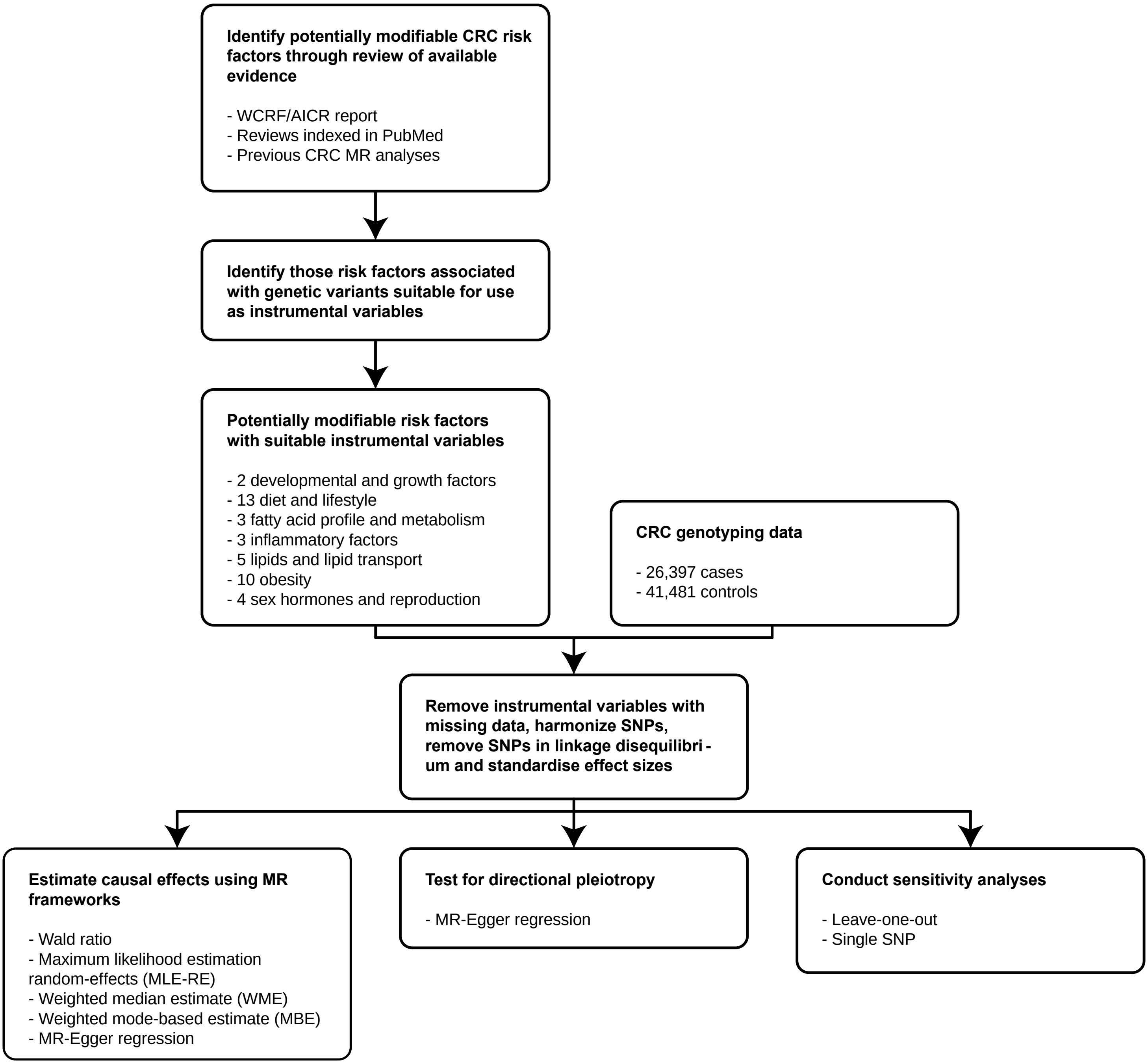

Supplementary Figure 1: Study design flowchart. CRC: colorectal cancer; MR: Mendelian randomization; SNP: single nucleotide polymorphism.

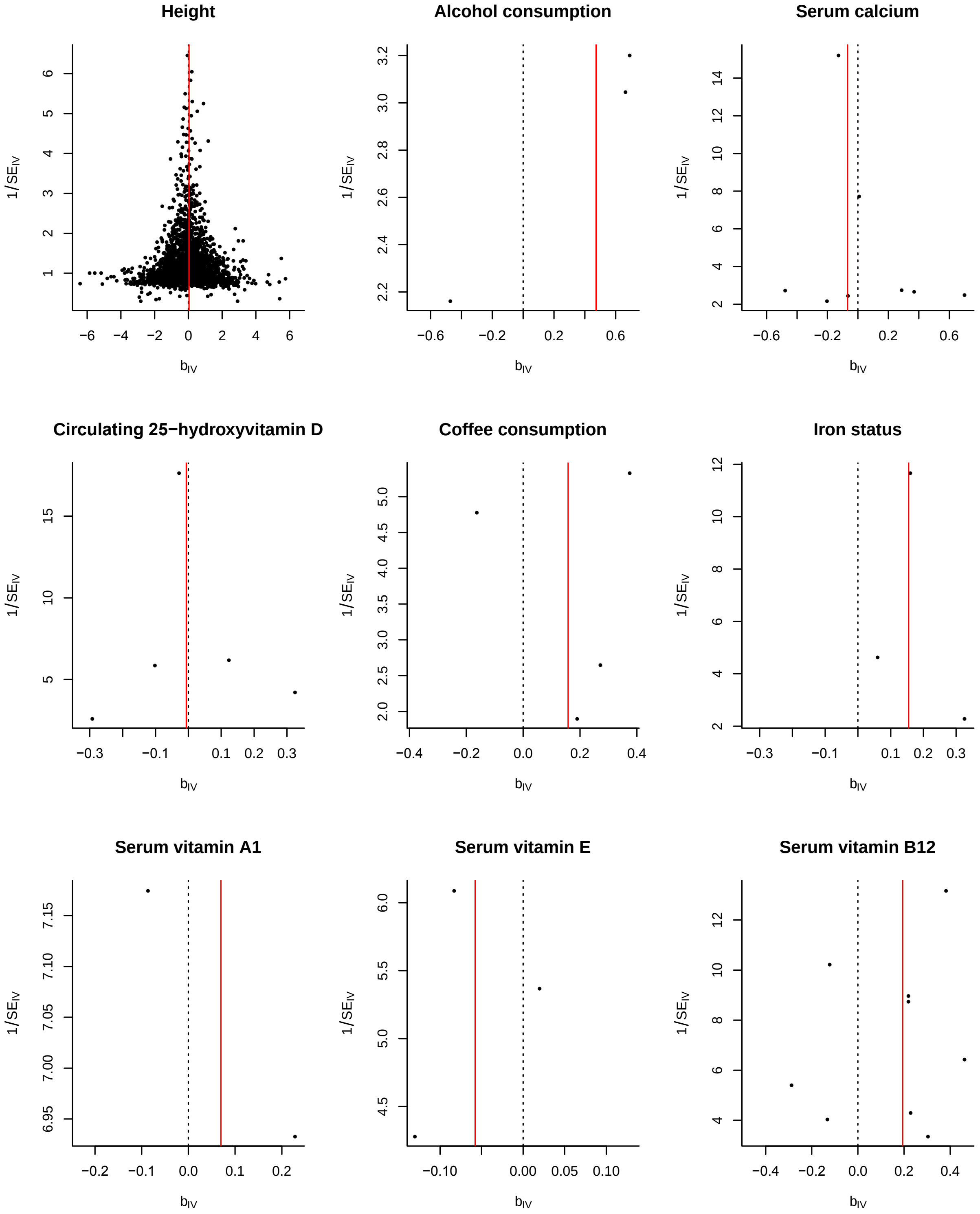

Supplementary Figure 2 (Page 1/4)

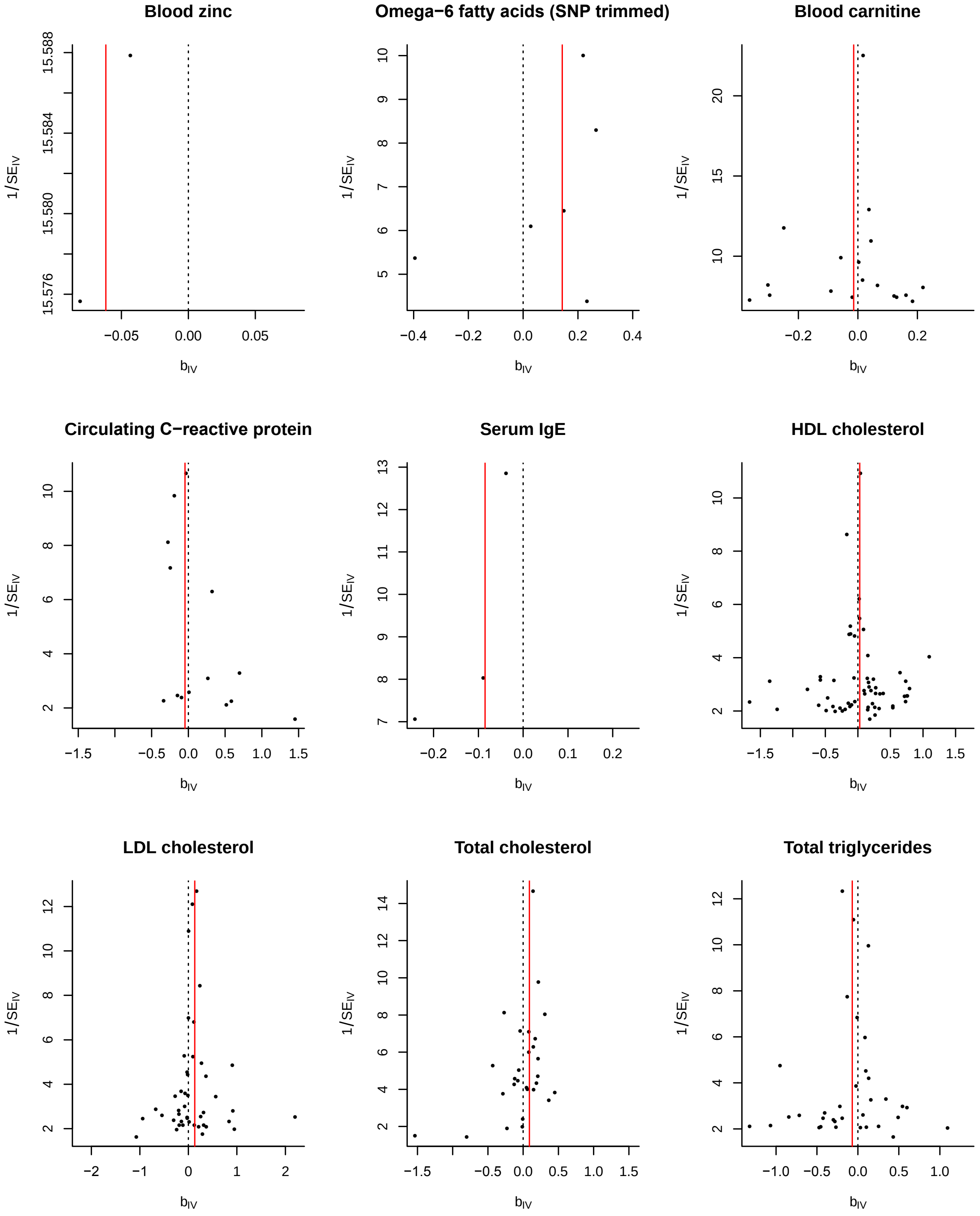

Supplementary Figure 2 (Page 2/4)

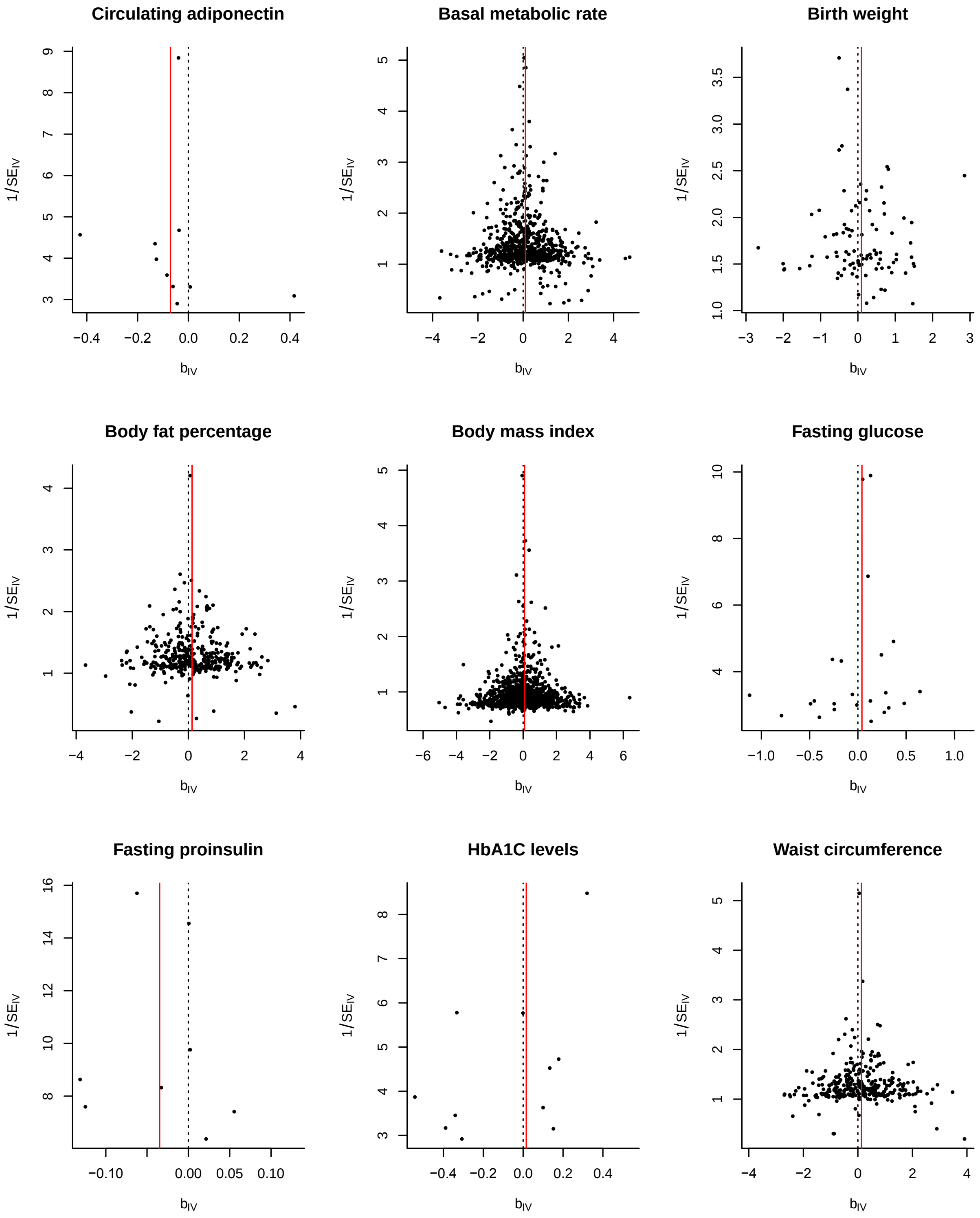

Supplementary Figure 2 (Page 3/4)

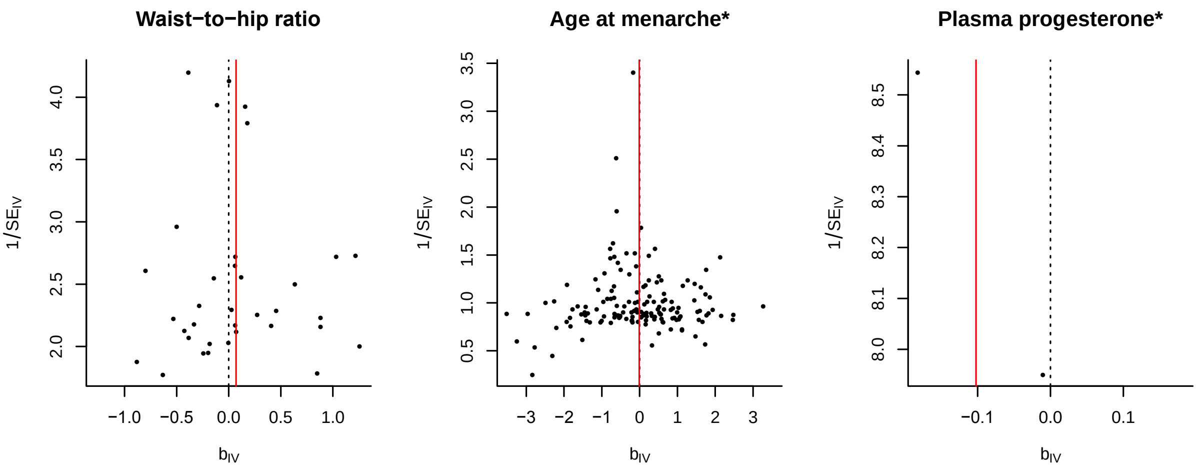

Supplementary Figure 2 (Page 4/4): Funnel plots of causal estimates (β_{IV}) and instrument strength ($1/SE_{IV}$) for each genetic variant used as an instrumental variable. Causal estimates computed as the log of the Wald ratio per genetically predicted standard deviation unit increase in the risk factor. Red lines represent causal effect estimated using a maximum likelihood estimate random-effects (MLE-RE) model. Dotted lines represent the null. SNP: single nucleotide polymorphism; HDL: high-density lipoprotein; LDL: low-density lipoprotein. *Causal effects estimated using colorectal cancer data from females only.

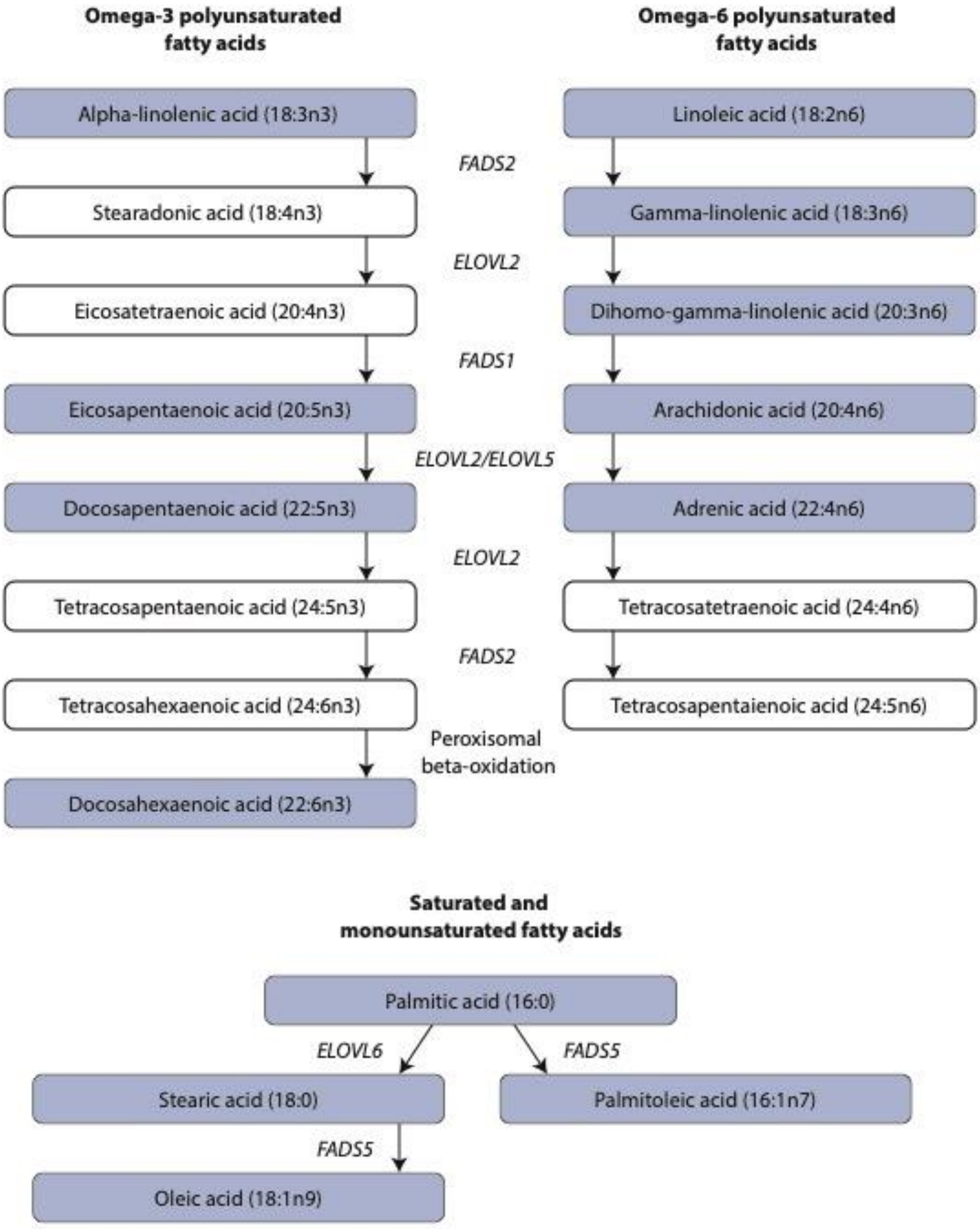

Supplementary Figure 3: Fatty acid pathways. Shown are the fatty acids considered in this MR analysis (coloured) and the genes encoding the enzymes catalyzing each pathway step.
